# Important India Rubber Case

**Published:** 1863-01

**Authors:** 


### IMPORTANT INDIA-RUBBER CASE.

UNITED STATES District Court, Southern District of New York. In equity. Henry B. Goodyear, as Administrator, and Conrad Poppenhusen *vs.* Meyer Dittenhoeffer, Joseph Steinert, Solomon Sonneborn, etc.; Storrs Brothers; Johnson, Byrne & Johnson; Rushmore, Cone & Co.; Chapman, Lyon & Noyes; George S. Murfey & Co.; Seligman, Rosenheim & Co.; Welton & Porters; Brown & Gray; Gerard H. Dwenger; Robert Haering, Leo Cohen & J. Rosenberg; Marks, Palmer & Cushman.

October 27, 1862.—Decree for complainants in the within suits, as prayed for in said bills, as in the case of complainants *vs.* New York Gutta-Percha and India-Rubber Vulcanite Company *et al.*

J. NELSON.

H. B. Goodyear, Administrator *et al. vs.* New York Gutta-Percha and India-Rubber Vulcanite Company, Oscar Falke *et al.*—Nelson, C. J.—The bill in this case was filed upon two re-issued patents to the complainants, as administrator of Nelson Goodyear, deceased, the original discoverer of new and useful improvements in the manufacture of India-rubber. The original patent to him bears date 6th May, 1851; the re-issued patents, 18th May, 1858. This invention relates to an improvement in the process of preparing India-rubber and other vulcanized gums, described in the previous well-known patent of Charles Goodyear, and by which improved process a new substance is produced, distinct in character from that produced by the invention of Charles Goodyear, and used for wholly different purposes. It is generally known as the hard rubber compound. The two re-issued patents claim, the one the process of manufacturing the hard rubber compound, the other the product. There was some doubt if the claim in the original patent embraced both these improvements, though fully described in the specification.

The utility of this improvement is not questioned. It has been before the Courts, incidentally, heretofore, and was the subject of observation. In the case of Poppenhusen *vs.* Falke and others, decided in October Term, 1861, on what is known as the "Tin Foil Patent" Judge Shipman, in delivering the opinion of the Court, observed, "that in the year 1851, Nelson Goodyear patented the peculiar substance known as the hard compound of india-rubber. He produced this remarkable material by combination, when operated on by the proper degree of heat, proved to be of great value, and well adapted to a great variety of uses. It is free from any disagreeable odor, impenetrable to ordinary fluids, hard, like ebony or ivory, susceptible of polish, and with an elasticity similar in kind to that of tempered steel. For many purposes of utility and ornament its value is proved by its extensive use in the community."

The only serious question arising out of the facts of this case is as to the originality of the invention by Nelson Goodyear. This has been strenuously contested by the learned counsel for the defendants, and requires some notice.

The proofs show that Goodyear began his experiments with a view to the improvement as early as the year 1847, and that he had nearly completed them as early as the summer of 1849. In December of that year he filed a caveat in the Patent Office, containing a description of the invention, and which embraces, substantially, all the information required at this day to manufacture the article.

This, as we have seen, was followed up by a patent dated 6th May, 1851—the application bearing date December, 1850.

The defense of want of novelty in Goodyear is placed mainly upon two grounds:—

1st. The patent of Austin G. Day, dated 9th November, 1858, and

2d. Patent to Charles Hancock, England, enrolled 11th July, 1846.

There are other criticisms in the proofs, and referred to in the argument of counsel, bearing upon the question; but we regard them as too unimportant to require any particular notice.

We have said that the patent of Austin G. Day has been relied on to show want of originality in Goodyear. Perhaps this is not quite an exact statement of the ground taken in the defense under that patent.

The defendants claim that they carry on their manufacture of the compound under this patent, and set up that the process is different from that of Goodyear.

The process of Goodyear is found in the caveat of December, 1849, the patent of May, 1851, and in the re-issues of May, 1858.

In the caveat he states: "My invention or discovery consists in the production, by means of a composition of India-rubber and sulphur subjected to intense heat, of a new and useful substance hitherto unknown, resembling, in hardness, bone or shell, but more extensively applicable, and less costly than either of those substances." "The main and indispensable ingredients of the composition are India-rubber, or caoutchouc, and sulphur. Of these I take certain proportions, say equal parts, by weight of each, and mix them thoroughly in any convenient manner. These proportions," he observes, "may, however, be considerably varied without changing materially the product."

Again he observes: "No precise rule of proportions can be given, or definite limits assigned, when sulphur alone is combined with rubber;" "that a much less quantity of sulphur than four ounces to a pound of rubber would be insufficient in any case."

In his patent of May, 1851, he observes: "Further experiments made by me since the filing of the caveat have confirmed the entire success of my invention."

And then, after describing the process—first, compounding India-rubber and sulphur, and, second, combining with them other ingredients—he states, "The proportions specified in both these compounds may be considerably varied without materially changing the result, but in no case will a much less quantity than four ounces to every pound of caoutchouc be sufficient, in which respect, particularly, my compounds differ very essentially from every other composition of India-rubber in use; as in all rubber compositions the least quantity of sulphur that will suffice to cure the article is aimed at."

The description in the re-issued patents in more full, and in greater detail, but substantially the same; the difference consists in separating the claim and issuing a patent for each.

Now Austin G. Day's patent is dated 9th November, 1858, nine years after the filing of Goodyear's caveat, and seven after his patent, and in his specification he says: "I took up the hard rubber manufacture at the time of the issue of the Nelson Goodyear patent of 1851, having a single object in view—namely, the manufacture of a hard, elastic compound, in which I used with success both rubber and gutta-percha."

Again he says: "My invention consists in a special mode of making hard, but highly elastic gum compound, by a process differing in length of time, in the degree of heat, and in the proportion of the ingredients, and in the mode of equalizing the temperature, from that prescribed by Nelson Goodyear."

He then claims—1st. "The running the heat for vulcanizing flexible and elastic hard-gum compounds through the high range of temperature, and the comparatively great length of time, substantially as set forth; that is to say, commencing the heat at about 275 degrees, and carrying the same to 300 degrees and upwards, substantially as described.

2d. "The making, as described, the flexible and elastic hard-gum composition of two parts, by weight, of rubber other vulcanizable gum, and one part sulphur, when such composition is preparatory to running of the heat, as described in these specifications."

Now, as the degree of heat to be applied in the manufacturing of this hard compound, Nelson Goodyear, in his patent of May, 1851, refers to the patent of Charles Goodyear of 1844, re-issued 1849, which gives a range from 212 degrees to 350 degrees, depending on the thickness of the composition, and adds: "In most cases the heat will be required to be raised as high as 260 degrees or 275 degrees, and the time of exposure of heat will range from three to six hours, or longer." The caveat stated the range between 250 degrees to 300 degrees, depending upon size of compound.

It is quite apparent, from this reference to the several patents, that there is nothing on the subject of the degree of heat in manufacturing the hard compound, described in the Austin patent, but what is found in that of Nelson Goodyear.

Then as to the proportions of India rubber and sulphur, Austin adopts two parts rubber and one sulphur.

Goodyear, in his patent of 1851, states that the best proportion will be about equal parts. That the proportions may be considerably varied without materially changing the result; but that in no case should it be less than four ounces of sulphur to a pound of rubber. The caveat contained the same substantially.

We perceive no ground for the claim to any improvement by Austin in this branch of the case.

Much was said on this argument, and also by witnesses on the part of the defendants, in respect to the qualities imparted to the hard compound by the patentees in the descriptions.

Goodyear, in his patent of 1851, claims the "combining of India rubber and sulphur, etc., for making a hard and inflexible substance, hitherto unknown, substantially as described."

In his caveat he describes it a hard and stiff substance, resembling in some respects horn or bone, and adapted to similar or more extensive uses.

Austin, in his patent, describes the article "flexible and elastic hard gum composition."

Now, it is apparent, from our previous examination of the patents of the respective parties, that this is simply a dispute about terms or words. The proofs also establish the same. An article of the same description of the Hard Rubber compound, and of the same qualities, was manufactured under the Goodyear patent from the time it was granted, as is made under the Austin patent, and, we may add, made under the former patent in Goodyear's factory, by Austin himself, who was in the service of the establishment. The idea of the learned counsel for the defendants seems to be that the patentee is bound, by the qualities imparted to the article, rather than by the qualities of the articles, as derived from the product of the compound patented. It would be a waste of time to attempt the refutation of so plain an error.

The main ground to establish the defense of the want of novelty in Goodyear's, is the English patent of Hancock, 11th July, 1846.

It is quite apparent, on anspection of this patent, that the patentee had not carried his experiments so far as to have produced the hard rubber compound of Goodyear, nor, as is obvious, had he any distinct or practical knowledge of it.

His combination or composition to produce the article is found in the third paragraph of the patent, which is a combination of India rubber or gutta percha, "with orpiments. liver of sulphur, or other sulphuret having like chemical properties," and he gives one general rule of proportion of orpiment or other sulphuret to be used, not to exceed twenty-five per cent. as applicable to the compound of all the products, whether soft or hard.

But what is more decisive is this: He observes that, "in making any of these compounds, a portion of sulphur may be used in place of an equal portion of orpiment or sulphuret; but I consider the use of the sulphur to be objectionable, because of the offensive smell which it imparts to the article, and, of the tendency which sulphur has to effloresce or exude from the surface."

No such consequence results from the compound of Goodyear's. On the contrary, as already stated, "it is free from any disagreeable odor, impenetrable to ordinary fluids, like ebony or ivory, susceptible of polish, and with an elasticity similar in kind to tempered steel."

This view is confirmed by a reference to a letter patent of Hancock, enrolled 10th August, 1847. In this he refers to his previous one, and observes that in the specification there he had recommended the sulphuretting of gutta percha should be effected by means of sulphurets, such as orpiment or liver of sulphur in preference to sulphur itself, etc. "I have since, however, ascertained that if a very minute portion of sulphur be used along with the sulphuret, a better result is obtained from a combination of the two, than from either substances alone."

It is clear that even in 1847, after the experiments of Goodyear had begun, that this patentee had not obtained any distinct idea of the American hard rubber compound.

Without pursuing the case further, we are satisfied that the defense fails, and that the complainants are entitled to the decree. NELSON, Judge.

### DECREE.

This cause having been brought to a final hearing upon the pleading and proofs, and counsel for the respective parties having been heard, and the same having been duly considered by the court, it is found hereby ordered, adjudged and decreed, that the two reissued letters patent, Nos. 556 and 557, granted unto Henry B. Goodyear, as administrator of the estate of Nelson Goodyear, deceased May 18, 1858, upon the surrender of the patent granted unto Nelson Goodyear, May 6, 1851, are good and valid patents, being the patents referred to in the complainants' bill, and that the said Nelson Goodyear was the original and first inventor of the improvements described and claimed in the said patents; and also that the said defendants have infringed upon the said patents, and upon the exclusive rights of the complainants under the same.

And it is further ordered, adjudged and decreed that the complainants do recover of the defendants the profits, gains and advantages which the said defendants, or any or either of them, have received or made, or which have arisen or accrued to them, or either of them, from said infringements of the said patents, by the manufacture, use, or sale of the improvements described and secured by the two reissued letters patent, Nos. 556 and 457, granted May 18, 1858, since May 18, 1858.

And it is further ordered, adjudged, and decreed that the said complainants do recover of the defendants their costs and charges and disbursements in this suit to be taxed.

And it is further ordered, adjudged and decreed, that it be referred to Kenneth G. White, one of the Masters of this Court, residing in the city of New York, to ascertain, and take, and state, and report to the Court an account of the gains, profits and advantages which the said defendants, or either of them, have received, or which have arisen or accrued to them or either of them, from infringing the said exclusive rights of the said improvements, patented in said reissued letters patent, May 18, 1858, since May 18, 1858.

And it is further ordered, adjudged and decreed, that the complainants, on such accounting, have the right to cause an examination of said defendants, and each of them *ore tenus*, or otherwise, and also the production of their books, vouchers, and documents of each of them, and that the said defendants attend for such purpose, before said Master, from time to time, as said Master shall direct.

And it is also further ordered, adjudged and decreed that a perpetual injunction be issued in this suit against the said defendants, according to the prayer of the bill.

KENNETH G. WHITE, Clerk.

